# A longitudinal qualitative study on the experiences and needs of women with gestational diabetes regarding blood glucose management using a journey map

**DOI:** 10.3389/fendo.2026.1866055

**Published:** 2026-06-17

**Authors:** Qiong Gao, Yuxia Yang, Lili Zhu, Cimei Li, Fuqin Zhang, Minghao Zhang

**Affiliations:** 1 Delivery Room, Xinxiang Central Hospital, Xinxiang, Henan, China; 2 Department of Reproductive Medicine, Xinxiang Central Hospital; Xinxiang Key Laboratory of Lifecycle Health Management and Humanistic Care Xinxiang, Xinxiang, Henan, China; 3 School of Nursing, Henan Medical University, Xinxiang, Henan, China; 4 Department of Obstetrics, East District Hospital, Xinxiang Central Hospital, Xinxiang, Henan, China

**Keywords:** gestational diabetes, longitudinal qualitative study, nursing, patient journey map, self-management

## Abstract

**Introduction:**

Gestational diabetes mellitus (GDM) is a common pregnancy-related metabolic disorder that increases risks of adverse maternal and fetal outcomes, as well as long-term type 2 diabetes. Effective glycemic management requires addressing women’s dynamic experiences and needs across pregnancy and the postpartum period. However, current evidence lacks longitudinal insights into patients’ real-world experiences and unmet needs in glycemic self-management. This study aimed to explore the longitudinal experiences and needs of women with GDM regarding blood glucose management using a patient journey mapping approach, to inform person-centered care models and improve self-management outcomes.

**Methods:**

A longitudinal qualitative design was conducted. Eighteen women with GDM were recruited from a tertiary hospital in Xinxiang, China, between September 2025 and March 2026. Semi-structured interviews were performed at three time points: diagnosis (24–28 weeks’ gestation), antenatal follow-up (3 months post-diagnosis), and postpartum follow-up (6 months post-diagnosis). Data were analyzed using qualitative content analysis, and a patient journey map was constructed covering tasks, pain points, and emotional dimensions.

**Results:**

Nineteen themes and subthemes emerged across three phases. During the diagnostic phase, women experienced emotional distress, knowledge gaps, and lifestyle adaptation difficulties. In the antenatal phase, self-management behaviors became internalized, accompanied by persistent vigilance, fatigue, and declining social support. Postpartum, women faced self-management burnout, role conflicts, and challenges in long-term glycemic control. The journey map visually depicted dynamic changes in emotions, tasks, and pain points throughout the continuum.

**Discussion:**

Glycemic management in GDM is phased and long-term, characterized by evolving experiences and needs. A tiered, nurse-led healthcare system integrating family support and *“*Internet+*”* technologies is recommended to deliver stage-specific interventions. Strengthening multi-source support and self-efficacy may reduce burnout and promote sustained self-management. These findings provide evidence for optimizing comprehensive care pathways for women with GDM.

## 1 Introduction

Gestational diabetes mellitus (GDM) is a prevalent metabolic disorder that manifests during pregnancy (1), with a global prevalence ranging from 1% to 14% (2). In recent years, there has been a marked upward trend in the incidence of the condition in China. GDM has been demonstrated to increase the risk of adverse pregnancy outcomes, including but not limited to gestational hypertension and caesarean delivery. In addition, GDM has been shown to be a contributing factor to fetal intrauterine growth restriction and neonatal hypoglycaemia. Furthermore, women with GDM have a significantly higher risk of developing type 2 diabetes after childbirth (3). Effective glycemic management exerts a direct influence on blood glucose control and maternal-fetal outcomes, while concomitantly reducing the incidence of adverse pregnancy outcomes and enhancing long-term prognosis (4). However, clinical practice has demonstrated that the glycemic management behaviours exhibited by women with GDM manifest in distinct phases, and there is a general absence of adherence to management protocols. Some patients struggle to maintain consistent glycemic management behaviours over the long term due to insufficient knowledge and a lack of support (5). The majority of extant studies (6–8) concentrate chiefly on decision-making behaviours, influencing factors, predictive models, or the short-term effects of interventions during the glycemic management process for GDM patients. A paucity of studies (9) has hitherto been published on the glycemic management experience of GDM patients. The extant literature consists of cross-sectional studies which lack longitudinal tracking of patients’ actual experiences and dynamic adaptation trajectories. Patient journey maps, when employed as a visualisation tool, have been shown to offer a clear illustration of patients’ experiences, needs, and challenges throughout the course of their illness, thereby providing precise evidence for phased nursing interventions (10). Therefore, this study aimed to explore the longitudinal experiences and needs of women with GDM regarding blood glucose management, so as to provide a theoretical and practical basis for optimizing comprehensive care models and improving glycemic management outcomes (11).

## 2 Methods

### 2.1 Ethical considerations

This study has been approved by the hospital’s Institutional Review Board (IRB): 2024-175-01(K). All participants voluntarily enrolled in the study and signed informed consent forms. It is imperative to note that all written records and audio recordings are intended solely for use in this study and may not be used for any other purpose or disclosed without the patients’ consent. The anonymity of participants has been preserved in the reporting of results.

### 2.2 Study design and participants

This study employed a longitudinal qualitative research design. The study employed purposive sampling to recruit women with gestational diabetes mellitus (GDM) who attended the obstetrics outpatient clinic at a Grade A Level 3 hospital—the highest tier of tertiary hospitals in China, providing comprehensive, specialized, and advanced medical services in Xinxiang City and underwent regular prenatal check-ups between September 2025 and March 2026. In order to ensure maximum diversity within the sample, a purposive sampling method was employed, with pregnant women of varying ages, educational backgrounds, and living situations being selected for inclusion in the study. The sample size was determined based on the principle of data saturation (12).

### 2.3 Inclusion and exclusion criteria

In order to ensure the rigour of this study, the following inclusion and exclusion criteria were established:

Inclusion criteria: ① Age ≥ 18 years; ② Diagnosis of GDM based on a 75g oral glucose tolerance test (OGTT) conducted between 24 and 28 weeks of gestation (13). Exclusion criteria: ① Presence of severe pregnancy-related complications or comorbidities; ② Cognitive impairment preventing completion of the questionnaire; ③ Presence of severe organic diseases. Culling criteria: ① Failure to complete at least two valid interviews at the scheduled interview time points; ② Request to withdraw from the study during the study period.

### 2.4 Data collection

First, Based on the research objectives, we initially developed an interview outline by reviewing the literature (14, 15), consulting experts, and analyzing the core elements of patient journey maps. We then conducted preliminary interviews with three pregnant women with gestational diabetes who met the inclusion criteria to refine the outline. The semi-structured guide is presented in **Table 1**.

**Table 1:** Semi-structured interview guide.

Theme	Interview questions
Understanding of GDM	1. How do you view your GDM diagnosis?
Self-management experience	2. How have you managed your blood glucose since diagnosis? 3. What challenges or feelings have you had during self-management?
Support needs	4. What kind of support do you need most? 5. In what form would you prefer to receive this support?

Second, Data were collected using semi-structured interviews conducted by a nursing graduate student with experience in blood glucose management for patients with gestational diabetes and training in qualitative research. This study employed a longitudinal design, and data collection at three time points was determined based on clinical trials, literature reviews, and expert discussions (16). The first interview (at the time of GDM diagnosis) was conducted face-to-face in a quiet outpatient office between 24 and 28 weeks of gestation; the second interview (during the antepartum period) was conducted face-to-face in a quiet outpatient office three months after diagnosis; and the third interview (during the postpartum follow-up period) was conducted via telephone or WeChat six months after diagnosis. Data collection strictly adhered to qualitative research standards. Prior to formal interviews, the researcher explained the study’s purpose, process, and significance to the pregnant women, informed them of their right to withdraw at any time, and recorded the sessions after obtaining informed consent. During the interviews, the researcher verified and confirmed certain viewpoints or open-ended responses from the women, while carefully observing and documenting their nonverbal behaviors. Each interview lasted 20–35 minutes.

Third, After the interviews concluded, two researchers jointly reviewed the audio recordings within 24 hours and transcribed them into text, while also organizing nonverbal information. Any points of uncertainty were clarified with the interviewees, and the participants were coded sequentially as F1 through F18 according to the order of the interviews.

Finally, The following steps have been proposed for the mapping of the patient journey (17, 18). The horizontal axis is divided into three time periods: the GDM diagnosis phase (24–28 weeks of gestation), the pre-delivery phase (3 months after diagnosis), and the postpartum follow-up phase (6 months after diagnosis). Utilising an observational approach, two senior obstetric nurses conducted dynamic, continuous observations of three GDM patients, meticulously documenting their self-management behaviours, key touchpoints between medical staff and patients, psychological and emotional changes, as well as nursing challenges. It is evident from the observations made that the task axis should be defined to include such elements as emotions, tasks, behaviours, pain points, and relevant personnel. The results of the interviews were analysed and integrated to systematically map the trajectory of behavioural changes in GDM patients’ self-management capabilities. This trajectory was then visualised using a journey map. In order to guarantee the comprehensiveness and consistency of the map’s content, it was subject to a joint review and refinement process involving researchers, patients, physicians, and primary caregivers.

### 2.5 Data analysis

The analysis was conducted using content analysis (19). The data was subjected to repeated reading, with meaningful language being identified and annotated. An independent review of the text was then conducted, with codes being compared and final codes being determined after reaching consensus. The final codes were then categorised and analysed based on the timeline of the patient journey map and the three core dimensions. The task dimension includes action verbs, process nodes, and tool usage. The emotional dimension includes patients’ emotional experiences, changes in psychological state, and contradictory statements. The pain point dimension includes barriers, unmet needs, and persistent concerns. The codes were then subjected to cluster analysis to distil themes and subthemes. The process was repeated until thematic saturation was achieved. Following an independent analysis of the themes at each time point, the results should be integrated for longitudinal comparison in order to identify commonalities and differences across different nodes. This will illustrate the dynamic changes in patient experience and needs (20).

### 2.6 Rigor

This study strictly adhered to the rigorous standards of qualitative research, ensuring reliability through every stage of the research process, including research design, data collection, analysis, and quality control (21). Participants were selected using purposive sampling, with patients with gestational diabetes (GDM) at different gestational ages and disease severities chosen to ensure the representativeness of the sample; Data collection utilized semi-structured in-depth interviews, which were fully audio-recorded and transcribed into written records; the transcripts were cross-checked repeatedly to ensure data authenticity. The analysis employed content analysis, with two researchers conducting independent coding and cross-checking, and the coding results were calibrated through expert consultation to minimize researcher bias. Methods such as member checking and triangulation were employed to enhance the credibility and validity of the research findings, ensuring that the conclusions are scientifically sound and reliable (22).

## 3 Results

The study ultimately enrolled 18 patients with gestational diabetes who met the inclusion criteria, and they were designated as F1 through F18. The demographic characteristics of the study participants are delineated in **Table 2**.

**Table 2:** The participant’s demographic characteristics (n=18).

Number	Age (years)	Educational level	Occupation	Residence	History of gestational diabetes	Parity	Gravidity	Conception method
F1	25	Associate degree	Unemployed	Town	No	G1P0	Twins	Natural conception
F2	32	Undergraduate	Doctor	Urban	No	G1P0	Twins	Manual
F3	27	Postgraduate	Teacher	Urban	Yes	G1P0	Single	Natural conception
F4	30	Postgraduate	Lawyer	Urban	No	G1P0	Single	Natural conception
F5	32	Undergraduate	Individuality	Urban	Yes	G2P1	Single	Natural conception
F6	37	Undergraduate	Staff	Town	No	G2P1	Single	Natural conception
F7	35	Associate degree	Teacher	Urban	No	G2P1	Single	Natural conception
F8	28	Associate degree	Staff	Urban	No	G1P0	Single	Natural conception
F9	30	Junior college	Unemployed	Rural	No	G4P1	Single	Natural conception
F10	28	Junior college	Unemployed	Rural	No	G2P0	Single	Natural conception
F11	25	Associate degree	Worker	Urban	No	G2P1	Single	Natural conception
F12	26	Vocational High School	Unemployed	Rural	No	G1P0	Single	Manual
F13	35	Undergraduate	Staff	Urban	No	G2P1	Single	Natural conception
F14	30	Vocational High School	Unemployed	Rural	No	G5P1	Single	Natural conception
F15	32	Undergraduate	Staff	Urban	No	G2P0	Single	Natural conception
F16	37	Vocational High School	Engineer	Rural	Yes	G4P2	Single	Natural conception
F17	39	Vocational High School	Unemployed	Rural	No	G6P2	Single	Natural conception
F18	42	Undergraduate	Worker	Urban	Yes	G4P2	Single	Natural conception

### 3.1 Patient journey map

**Figure 1:**
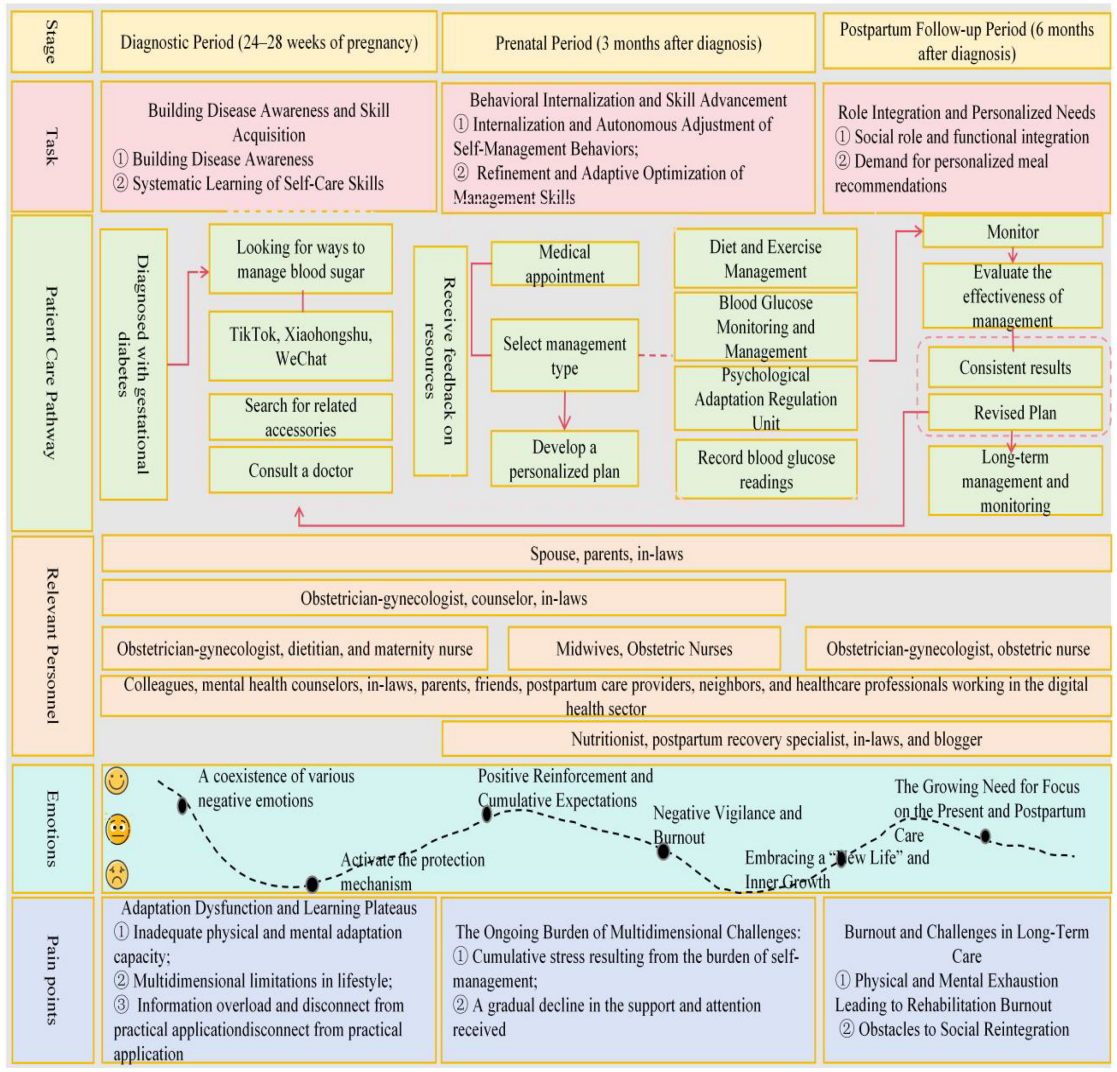
A journey map for blood glucose management in women with gestational diabetes.

This study involved conducting semi-structured, in-depth interviews with patients diagnosed with gestational diabetes (GDM) who met the inclusion criteria during diagnosis, labour and the postpartum follow-up phases. Using a patient journey map as a framework, the study explored patients’ experiences with blood glucose management and their evolving needs across different stages of the disease, focusing on three key dimensions: emotional experiences, core tasks, and key pain points. Ultimately, 19 themes and subthemes were identified, as shown in **Figure 1**.

### 3.2 Diagnostic period: emotional imbalance and self-management difficulties due to cognitive deficits

#### 3.2.1 Emotions: coexistence of multiple negative emotions, triggering protective mechanisms

##### 3.2.1.1 Coexistence of multiple negative emotions

When patients are confronted with their diagnosis without prior warning, they are likely to experience a range of negative emotions, including shock, denial, anxiety, and confusion.

F1: “I had never been acquainted with the term GDM prior to this. On the day of the diagnosis, a state of immediate panic was experienced. The condition was a cause for concern as it was suspected to have an impact on the infant’s development. The individual expressed a lack of clarity regarding the subsequent course of action and a need for more comprehensive explanations from the relevant parties.”

F2: “Upon receiving the diagnosis, I experienced a period of profound emotional distress that lasted for a considerable duration. I felt as though I had harmed my baby because I had not been vigilant in my dietary choices, and I held myself culpable to a great extent.”

F6: “The necessity to regulate my diet and monitor my blood sugar on a daily basis was a sudden and unanticipated imposition which left me at a loss as to how to proceed. As the subject of consideration was contemplated further, a state of increased anxiety was experienced. The subject expressed concern that difficulties would ensue if they were unable to regulate their blood sugar levels.”

##### 3.2.1.2 Activating the protection mechanism

The majority of patients encounter difficulties in accepting the diagnosis. The subjects then proceed to collate information, undergo various tests, consult doctors, and seek support from peers. This process is undertaken repeatedly in order to verify the accuracy of the diagnosis, thereby enabling them to cling to their remaining hope and activate their self-protection mechanisms.

F4: “Following the doctor’s diagnosis, I began to engage in persistent searching on Douyin and Xiaohongshu.”

F18: “We proceeded to visit multiple medical facilities in order to have the results of the aforementioned tests re-examined.”

F9: “I can confirm that I made enquiries of acquaintances who had received abnormal Down’s syndrome screening results but normal non-invasive DNA test results. This information served to heighten my level of optimism.”

Furthermore, patients may adopt avoidance behaviours, such as social withdrawal and distraction, in order to protect themselves.

F13: “I have been sequestered in my room for several days and refrained from communicating with others.”

F15: “I have been engaged in household chores incessantly in an attempt to distract myself from the matter.”

#### 3.2.2 Task: building disease awareness and skill acquisition

##### 3.2.2.1 Building awareness of the disease

While patients have a basic understanding of GDM, attempt blood glucose monitoring, adjust their diets, and engage in low-intensity exercise, most do not adhere to these tasks effectively and remain passive and uncertain.

F3: “The physician simply instructed me to regulate my blood sugar levels, but did not provide any specific guidance regarding dietary restrictions or physical activity recommendations.”

The present author is only able to reach a conclusion regarding this matter through independent means. In instances of time constraints, the subject reports omitting blood sugar checks, which engenders uncertainty regarding potential consequences.

F4: “I endeavour to consume a reduced quantity of starchy foods, yet I am uncertain as to what alternative sustenance I should ingest. On occasions when the subject experiences a craving for food, they will admit to surreptitiously consuming biscuits, a practice which is subsequently accompanied by a sense of remorse.”

F7: “I inquired with the physician as to whether I would be permitted to engage in physical activity, to which they replied in the affirmative, though they did not provide any specific guidance on the nature of the activity or its duration. I am reluctant to engage in physical activity for fear that it might have a negative impact on the foetus.”

##### 3.2.2.2 Systematic learning of self-defense skills

Patients must cultivate competencies to tackle challenges such as a paucity of knowledge concerning GDM diets, an absence of personalised guidance, and a dearth of avenues for addressing negative emotions.

F8: “A plethora of divergent information regarding GDM is present on my telephone. Some sources indicate that fruit consumption is permissible, while others suggest its restriction. The veracity of this information is unclear, and further elucidation from a medical professional has not been forthcoming due to time constraints. I feel both anxious and scared.”

F9: “My family members are not cognizant of GDM either. The subject expressed a desire to have someone with whom to discuss their concerns, but stated that this was not possible. Instead, they indicated that they had to keep their feelings to themselves, and that this made them feel worse.”

F12: “I am endeavouring to maintain a healthy diet, however my mother-in-law persistently encourages me to consume high-sugar and high-carbohydrate meals.”

#### 3.2.3 Pain points: adjustment difficulties and learning plateaus

##### 3.2.3.1 Poor physical and mental adaptation to stress

The presence of both unpredictable complications arising from the disease itself and pregnancy-related symptoms can result in significant physical discomfort and psychological distress for patients.

F13: “I was already suffering from morning sickness, and now, despite being able to eat whatever I want, I am reluctant to do so as I am afraid that the disease will put an extra strain on my body and be bad for the baby.”

F9: “In the context of the dinner table, when confronted with visually appealing and palatable culinary offerings, my immediate cognitive response is to reflect upon my present state of affairs, which effectively curtails any inclination to partake in the consumption of these items.”

F5: “GDM has a detrimental effect on my physical and mental well-being, to the extent that I am reluctant to disclose it to my friends.

##### 3.2.3.2 Multidimensional limitations on lifestyle

The disease necessitates strict adherence to a diabetic diet, exercise regimen, and social restrictions, thereby depriving patients of autonomy in their daily lives.

F1: “I am currently abstaining from the consumption of sugary foods, in order to avoid the potential for a rapid increase in blood sugar levels.”

F17: “Previously, I had a fondness for sweets and spicy food. However, due to my blood sugar issues, I have been compelled to modify many of my dietary habits.”

F5: “I invariably eschew socialising with friends as I am apprehensive that others may become cognisant of my limitations.”

F6: “I am no longer able to consume carbohydrates; I am only able to ingest whole grains because I am afraid that my blood sugar will rise.”

F8: “I previously enjoyed physical activity, however, I now refrain from intense exercise due to my apprehension regarding hypoglycaemia. It feels as if my life has been restricted.”

##### 3.2.3.3 Information overload and a disconnect from practical application

Written information and verbal instructions fail to translate into daily practice, leaving patients feeling confused and doubting themselves.

F3: “During my hospitalisation, I was provided with various informational booklets and brochures, and I attended educational sessions conducted by the nursing staff. However, the volume of information was overwhelming. I was unable to recall any of the relevant information, and upon returning home, I experienced a sense of disorientation and confusion, losing track of the tasks I was expected to perform.”

F6: “Following the ingestion of food, an attempt was made to engage in physical activity in accordance with the routine outlined by the nurse. However, despite dedicated practice, there was a persistent uncertainty regarding the accuracy of the movements. The efficacy of the routine was also called into question.”

F11: “The educational handbook provided by the physician was excessively general in nature, comprising solely broad principles. Despite extensive research, no specific plan tailored to my gestational age and weight was found.”

### 3.3 Pregnancy

#### 3.3.1 Emotions: a mix of conflicting emotions

##### 3.3.1.1 Positive reinforcement and cumulative expectations

The enhanced glycemic control has had a positive impact on the patients’ morale, fostering a sense of optimism regarding their recovery and the potential for a healthy pregnancy.

F1: “I feel much better now. This is indicative of the author’s progress towards achieving their ideal condition. I am confident that the infant will be in good health, and I am generally satisfied with the management of my condition.”

F8: “I can consume fruit as a snack during the day, and this is comparable to a normal pregnancy.”

##### 3.3.1.2 Negative vigilance and burnout

For some patients, the necessity to perpetually monitor blood glucose levels can precipitate persistent anxiety, and the anxiety engendered by blood glucose fluctuations, in conjunction with the fatigue that ensues from protracted blood glucose management, engenders a pernicious cycle.

F12: “I have gradually come to the understanding that, provided I manage my blood sugar levels effectively, a positive outcome will be assured, and I feel considerably more at ease. However, there are occasions when there is a sudden spike or drop in blood sugar levels, and this can give rise to concerns about its potential impact on the infant, particularly during prenatal consultations when feelings of anxiety are particularly pronounced.”

F13: “For a period of three months, I have been undertaking the management of my blood sugar levels. The necessity to undertake four blood sugar checks and meticulous portion calculations per day is a significant source of physical and mental fatigue. On occasion, the urge to satisfy cravings with a sweet treat arises, yet subsequent feelings of guilt ensue.”

F15: “The date of delivery is imminent. There are two factors to consider. Firstly, there is the potential impact of unstable blood sugar levels on the delivery process. Secondly, there is the possibility that ongoing management of blood sugar levels will be necessary postpartum. The more I reflect on it, the more anxious I become.”

#### 3.3.2 Task: internalization of behavior and skill advancement

##### 3.3.2.1 Internalization and autonomous regulation of self-management behaviors

It has been observed that some patients have gradually adapted to a diabetes-friendly diet and have internalised the schedule and portion control plan as part of their daily routine, adjusting it flexibly based on their personal experience and living circumstances.

F5: “I now adjust my portion sizes and eating habits based on my blood glucose readings.”

F16: “I have modified my dietary intake to consist of smaller, more frequent meals. This has enabled me to more effectively regulate my blood sugar levels.”

F6: “Historically, I adhered meticulously to the meal schedule that was recommended. However, I now find myself modifying this schedule in accordance with my own circumstances.”

F1: “I adhere to a vegetarian diet. The physician recommended an increase in protein intake, yet there is a paucity of information regarding which vegetarian foods are high in protein. There has been no provision of a specific meal plan, and so the subject is left to determine this information independently.”

##### 3.3.2.2 Refinement and adaptive optimization of management skills

Patients periodically adjust their diet and activity levels in accordance with the results of at-home fingerstick blood glucose and blood tests monitoring fetal growth, thereby advancing their self-management skills.

F15: “I frequently attend the hospital for ultrasonographic examinations to monitor foetal growth. Despite the doctor’s assurances regarding the infant’s well-being, I find myself unable to shake this concern.”

F11: “My former dietary regime was characterised by a high consumption of greasy foodstuffs and carbohydrates. However, I have since adopted a more nutritionally balanced diet, resulting in improved glycemic control. It is imperative that I undergo regular check-ups and adjust my meal plan in accordance with the results.”

#### 3.3.3 Pain points: the ongoing burden of multifaceted challenges

##### 3.3.3.1 The burden of self-management leads to cumulative stress

In order to prevent complications, it is imperative that women with GDM monitor their blood sugar levels and develop a regular exercise routine. This necessitates a degree of self-discipline that is distinct from that which is required during a standard pregnancy, thereby placing an increased burden on patients in terms of self-management.

F10: “At present, the primary focus is on the regulation of blood sugar levels, with daily checks and regular meals at set intervals. It is hoped that stability will be maintained until delivery, thus avoiding any interference from GDM in the process of a natural birth.

F14: “I have learnt some breathing techniques for labour online, and I frequently inquire with the midwife about the precautions that should be taken during delivery. I am concerned that complications might arise due to blood sugar issues.”

F13: “I did not encounter any issues with my first child. Therefore, it is perplexing to me why my blood sugar levels are elevated on this occasion. The physician has repeatedly emphasised the risks associated with GDM, which has led to an increased level of self-discipline on my part.”

##### 3.3.3.2 The support and attention received have gradually waned

It has been reported by some patients that, over time, the level of attention, reminders, and support provided by family and friends has diminished.

F4: “Initially, my husband was responsible for ensuring I adhered to the regimen, however, he has since ceased to provide me with the necessary support.”

F13: “My mother-in-law asserts that the infant’s growth is impeded without the consumption of substantial, fatty foods. Consequently, she asserts that we will regulate my diet during the subsequent visit.”

F16: “During follow-up visits, I occasionally inquire with the physician regarding the necessity of maintaining such stringent blood sugar control measures following childbirth. Furthermore, it is important to ascertain how to manage this issue postpartum in order to avoid a situation where necessary steps are not taken in a timely manner. However, the doctor’s attitude appears to be one of minimal concern.”

### 3.4 Postpartum follow-up period

#### 3.4.1 Emotions: growing focus on personal growth and postpartum care needs

##### 3.4.1.1 Embracing a *“*new life*”* and inner growth

In light of the transition in postpartum roles and the nature of the condition, some patients adopt a positive outlook on life, prioritising their own recovery and the well-being of their infants.

F7: “Overall, I feel as though I am recovering steadily. However, some patients express concerns that their blood sugar levels will not return to normal, while others neglect self-management due to their preoccupation with caring for their infants.”

F17: “My days are entirely consumed by the needs of my infant, including feeding and diaper changes, which leaves no time for blood sugar monitoring. It was hypothesised that the transition to motherhood would be accompanied by a relinquishment of these vices, with occasional indulgences in confectionary being permitted without significant remorse.”

##### 3.4.1.2 The growing need for focus on the present and postpartum care

As patients undergo the process of childbirth, the majority typically experience a gradual return to normal blood sugar levels, accompanied by the establishment of consistent self-management routines. However, the demands of infant care and physical recovery present new challenges.

F12: “Fortunately, I have been able to maintain optimal blood sugar levels. A child in the parish was recently referred to the paediatric department for elevated blood glucose levels. This experience has instilled in me a profound sense of gratitude for life itself.”

F5: “Despite the fact that I have hitherto managed it well, I still occasionally worry about the possibility of developing diabetes. In such circumstances, I endeavour to engage in distracting activities in order to avoid dwelling on the issue.”

F9: “My blood sugar levels have not yet returned to normal following childbirth. The patient is characterised by a state of perpetual anxiety, which is acutely experienced during follow-up visits. The patient’s concerns primarily revolve around the uncertainty surrounding the duration of self-managed blood sugar levels and the potential for more efficacious management strategies.”

F8: “Despite experiencing a sense of relief following the birth of the infant, I continue to experience feelings of anxiety each time I undertake blood sugar monitoring. I am concerned that I will not recover adequately and that I may subsequently develop diabetes, which could in turn compromise my ability to breastfeed.”

#### 3.4.2 Task: role integration and personalization needs

##### 3.4.2.1 Integration of social roles and functions

Once their physical condition has stabilized, patients attempt to reintegrate into their previous family and social roles and gradually return to their normal lives.

F17: “I want to manage my blood sugar properly, but as soon as my child cries, I can’t focus on anything else. Sometimes I don’t even check my blood sugar once a day, and exercising is out of the question.”

F7: “As a good mother, I need to breastfeed my child more often to ensure they get proper nutrition, so I can’t keep up with my blood sugar.”

F10: “I want to go back to work, but my family wants me to rest and take care of the child.”

F18: “I’d love to join a WeChat group with doctors and other women with gestational diabetes. That way, I could ask questions right away when I have them and share experiences with others to help relieve some of the emotional stress.”

##### 3.4.2.2 Requirements for personalized recipe recommendations

Patients have expressed a keen interest in receiving bespoke dietary recommendations.

F4: “It is my hope that the dietitian will be able to devise a meal plan that is tailored to my particular physical condition. For instance, I am currently suffering from anaemia, and as such I am keen to ascertain which foods would be most beneficial for my condition.”

F16: “I am keen to make adjustments to my diet, however, given that I am breastfeeding, I am concerned that a reduction in the consumption of carbohydrates may have a negative impact on my milk supply. While I am able to attempt to reduce my consumption of confectionary, I am uncertain as to whether this is the most appropriate strategy.”

F14: “During my postpartum checkup, the physician advised me to engage in more physical activity. However, I am experiencing a period of considerable weakness and am responsible for caring for my infant. The opportunity for physical activity is limited to the hours spent asleep by the infant, which precludes adherence to the recommended duration stipulated by the doctor.”

F10: “The necessity for guidance on a personalised dietary and exercise regime, in conjunction with advice that integrates postpartum recovery with blood sugar management, is paramount. The guidance I am currently receiving is excessively generic and does not align with my particular circumstances.”

#### 3.4.3 Pain points: burnout and challenges in long-term management

##### 3.4.3.1 Physical and mental exhaustion leading to rehabilitation burnout

The physical strain of long-term breastfeeding after childbirth, in combination with the mental and physical exhaustion resulting from strictly following medical advice, has led to a complete depletion of the patient’s psychological resilience. This, in turn, has resulted in a reduced adherence to self-management.

F12: “I am obliged to rise at a consistent hour each day in order to breastfeed, and I experience a considerable degree of pressure as a result.”

F10: “During the postpartum confinement period, it is recommended that I consume substantial quantities of soups and broths to ensure a sufficient milk supply. However, this results in rapid spikes in my blood sugar levels, which creates a challenging dilemma for me.”

F16: “My body is quite weak after giving birth. The subject has expressed a desire to perform rehabilitation exercises; however, they have expressed concern that these may have a negative impact on their blood sugar levels. In the course of my enquiry, I sought clarification from the doctor on the most effective method of balancing the two, but they were unable to provide a definitive response. This situation is deeply concerning. It is hoped that a healthcare professional specialising in this area will be able to provide adequate guidance on the management of blood sugar levels, thereby facilitating a successful recovery.”

##### 3.4.3.2 Barriers to social reintegration

In the aftermath of childbirth, patients often encounter difficulties when attempting to resume their professional and social responsibilities. These difficulties may manifest in a range of challenges, including a sense of emotional detachment and a struggle to adapt to their professional environment and new roles.

F15: “The necessity to order takeout following my return to work presents a significant challenge in the effective management of my blood sugar levels.”

F9: “I have been on leave for a considerable period of time, which has led to concerns regarding my ability to resume my professional duties. Additionally, I am apprehensive about the possibility of being marginalised once I return to work.”

F11: “I have not been professionally active for a considerable period of time, and I am uncertain as to whether I possess the necessary capabilities to fulfil the responsibilities of the position.”

## 4 Discussion

### 4.1 Establish an “internet +” healthcare management system led by specialized nurses and supported by family involvement to improve access to blood glucose management

This study reveals a persistent mismatch between routine nursing services and the real needs of women with GDM across all disease stages. Challenges including information overload, poor translation of theoretical guidance into daily practice, lack of personalized instruction, declining social support, and heavy childcare responsibilities collectively hinder sustained glycemic self-management. These observations align with previous research indicating that standard clinical education is often generic and fails to address individual circumstances and contextual barriers throughout the care continuum (23–25).

Consistent with existing evidence, family involvement emerges as a critical but underutilized resource. Systematic family education can transform caregivers from passive observers into active participants, assisting with blood glucose monitoring, dietary planning, and emotional encouragement—all of which are known to improve self-management adherence (8). Additionally, the “Internet+” model, via WeChat and mobile mini-programs, enables real-time consultation, remote follow-up, and peer interaction, effectively overcoming geographical and temporal limitations and complementing offline care (26). Integrating these elements, a nurse-led healthcare system supported by family participation and “Internet+” technology can deliver stage-specific, targeted interventions, reduce uncertainty, and enhance sustained engagement throughout the GDM care trajectory (27, 28).

### 4.2 Mobilizing internal and external support systems to enhance self-regulation in women with gestational diabetes

This study demonstrates that, during the postpartum follow-up period, patients with GDM experience reduced adherence to blood glucose management due to burnout, leading to challenges such as a lack of commitment to recovery and psychological barriers to social reintegration. Furthermore, their self-regulation abilities are highly correlated with the adequacy of their subjective and objective support systems, and the two factors form a mutually reinforcing feedback loop. On the one hand, the cumulative fatigue resulting from long-term blood glucose management and concerns about maternal and infant outcomes leads to self-management burnout (19, 29), creating a potential barrier to the rehabilitation process. Conversely, patients encounter participation barriers that stem from the transition to maternal roles, workplace adaptation, anxiety about postpartum body image, and potential stigma associated with the condition. These barriers impede their ability to seamlessly reintegrate into social life, a finding consistent with research on the sense of social isolation among GDM patients (30). Strict maternal glycemic control is closely linked to maternal and infant birth outcomes. Poor blood glucose management significantly raises risks of macrosomia, preterm birth, cesarean section, gestational hypertension and prolonged labor, and also increases the occurrence of neonatal hypoglycemia and respiratory complications (31). In contrast, steady glycemic regulation can effectively reduce adverse delivery events and safeguard perinatal safety of both mothers and infants. Optimized multi-source support helps maintain stable blood glucose levels, which in turn greatly improves overall birth outcomes. The maintenance of stability in the subjective support system is contingent upon continuous psychological empowerment and cognitive enhancement, thereby fostering sustained self-regulation capabilities (20). Therefore, it is recommended that healthcare providers establish a three-tiered support network system. Firstly, a multidisciplinary team should be formed, centred on medical and nursing expertise, with the utilisation of specialised nurses to provide targeted care. The primary focus should be on optimising self-management strategies for patients with gestational diabetes (GDM). This should include the implementation of measures such as dietary management and blood glucose monitoring. It is also imperative to emphasise the impact of adherence on positive maternal and infant outcomes. Secondly, the emphasis should be on cognitive enhancement and psychological empowerment. This can be achieved by using scenario-based simulation training to improve family members’ collaborative capabilities and implementing interventions in phases. Furthermore, resources such as psychological counselling, peer support groups, and emotional management should be provided to assist patients in balancing blood glucose management behaviours with the gradual social reintegration associated with role transitions. This will facilitate the shift towards a long-term health management model and enhance their self-efficacy in reintegrating into society.

### 4.3 Implementing a tiered support system to promote the social reintegration and long-term management of patients with gestational diabetes

The postpartum period represents a critical transition phase where prolonged self-management burden, maternal role conflicts, and intensive childcare responsibilities converge, frequently leading to self-management burnout and reduced adherence. These challenges align with existing literature documenting that chronic disease self-care behaviors commonly decline after delivery, as maternal identity and infant care priorities dominate daily life (19, 29).

The findings of this study emphasize the necessity of establishing a tiered, phase-adaptive support system. First, multidisciplinary teams, centered on specialized nurses, should prioritize long-term metabolic risk education and regular postpartum follow-up, addressing persistent concerns about type 2 diabetes development and reinforcing the importance of sustained glycemic monitoring (20). Second, cognitive enhancement and psychological empowerment interventions are essential to help women balance postpartum physical recovery, infant care, and glycemic management goals, reducing emotional distress and role strain (30). Third, family-centered collaborative training and peer support networks can alleviate social isolation and facilitate smoother role transition (14). Such a structured tiered support system promotes successful social reintegration, sustains long-term self-management habits, and supports the transition to lifelong health monitoring—key to reducing the long-term risk of type 2 diabetes and other metabolic complications in women with a history of GDM.

This phased whole-cycle management model possesses strong universal applicability and can be widely promoted beyond the Chinese medical context without relying solely on WeChat platforms. In European, American and other Western countries, local mainstream instant messaging software, official health Apps, email consultation systems and hospital remote follow-up platforms can replace WeChat to realize online health education, regular follow-up, real-time question answering and peer group communication (19).

In Southeast Asia, Africa and other regions with relatively simple network conditions, basic mobile phone SMS notification, community offline health lectures and primary medical station home visits can be adopted to carry out simplified hierarchical support services. The core ideas including stage-based psychological intervention, family joint participation and long-term metabolic risk management are not limited by regional network environment and social media tools.

Regardless of differences in medical systems, dietary culture and living habits in various countries, the dynamic demand law of GDM women throughout pregnancy and puerperium is consistent. Researchers and clinical nurses in different countries can adjust specific implementation carriers and service forms according to local actual medical resources, cultural customs and public health service conditions, retain the core framework of this patient journey-based continuous management model, and formulate localized gestational diabetes mellitus nursing management schemes suitable for local crowds.

### 4.4 Strengths, limitations and future directions

#### 4.4.1 Strengths of the study

This study adopted a longitudinal qualitative research design, which effectively captured the dynamic changes in psychological status, self-management behaviors and actual needs of women with gestational diabetes mellitus at different disease stages, rather than only exploring cross-sectional static experiences. Combined with patient journey map for data sorting and visual analysis, the research results are more systematic and intuitive, which can better reflect the real clinical experience of participants. In addition, this study fully considers the actual medical environment and family living situation of participants, and the research findings have good practical guiding significance for clinical nursing intervention.

#### 4.4.2 Research limitations

First of all, all participants in this study were recruited from a single tertiary Grade A hospital in a single region, the research sample source is relatively single, which may affect the external generalizability of the research conclusions. Secondly, this study only included pregnant women with GDM as research objects, and did not collect the perspectives of clinical medical staff, family caregivers and other related groups, so the research perspective is not comprehensive enough. Thirdly, restricted by time and research conditions, the follow-up time was relatively short, and long-term follow-up data after postpartum were insufficient.

#### 4.4.3 Future research directions

In future studies, multi-center and multi-region sampling can be carried out to expand the research scope and further verify and enrich the research conclusions. Researchers can also incorporate the viewpoints of medical staff and family members to build a more complete research framework. On this basis, phase-oriented targeted nursing intervention programs can be formulated according to the phased needs summarized in this study, and further intervention effect verification can be carried out. Meanwhile, prolonged long-term follow-up investigation is suggested to explore the long-term glycemic control status and health outcome changes of women with GDM, so as to provide more sufficient evidence for establishing whole-cycle continuous management mode.

## 5 Conclusion

This longitudinal study clarified the dynamic emotional states, self-management behaviors and practical needs of women with gestational diabetes mellitus across diagnosis, antenatal and postpartum stages. Distinct phase-specific barriers were identified throughout the full care trajectory. Targeted multi-level support combining nurse-led intervention, family participation and internet-based health management is urgently required. Such tailored strategies can effectively relieve psychological distress, improve self-management adherence and ease role adaptation pressure. Furthermore, the established phased care framework is flexible and universally applicable. It can be adapted via local mainstream communication and health platforms rather than being confined to regional social tools, enabling wider promotion in diverse medical and cultural settings. In clinical practice, implementing whole-cycle staged nursing measures is conducive to optimizing glycemic control outcomes and reducing long-term metabolic risks among affected women.

## Data Availability

The raw data supporting the conclusions of this article will be made available by the authors, without undue reservation.

## References

[1] Bianco ME Josefson JL . Hyperglycemia during pregnancy and long-term offspring outcomes. Curr Diabetes Rep. (2019) 19:143. doi: 10.1007/s11892-019-1267-6 31754898 PMC7008468

[2] Gao C Sun X Lu L Liu F Yuan J . Prevalence of gestational diabetes mellitus in mainland China: a systematic review and meta-analysis. J Diabetes Invest. (2019) 10:154–62. doi: 10.1111/jdi.12854 29683557 PMC6319492

[3] Saravanan P . Gestational diabetes: opportunities for improving maternal and child health. Lancet Diabetes Endocrinol. (2020) 8:793–800. doi: 10.1016/S2213-8587(20)30161-3 32822601

[4] Kyto M Markussen LT Marttinen P Jacucci G Niinisto S Virtanen SM . Comprehensive self-tracking of blood glucose and lifestyle with a mobile application in the management of gestational diabetes: a study protocol for a randomised controlled trial (eMOM GDM study). BMJ Open. (2022) 12:e66292. doi: 10.1136/bmjopen-2022-066292 36344008 PMC9644362

[5] Lv Y Li Q Wu J Zhang X Gu R Pan Y . Mapping the health management journey of women with gestational diabetes mellitus: a qualitative study. BMC Pregnancy Childbirth. (2025) 25:1239. doi: 10.1186/s12884-025-08409-y 41257627 PMC12629020

[6] Sun K He F Zhang R Lu M Zhao J Kong J . Application progress of digital health technology in nutritional management of patients with gestational diabetes mellitus. Chin J Nurs. (2025) 60:1694–9. doi: 10.3761/j.issn.0254-1769.2025.14.005

[7] Wang Q Zhang K Zhang X Zheng Q Yang J Lin J . A scoping review on the application of mobile blood glucose management in pregnant women with gestational diabetes mellitus. Chin J Nurs. (2024) 59:1270–7. doi: 10.3761/j.issn.0254-1769.2024.10.017

[8] Zhang X Yin W Liu Q Jin H Zu H . A longitudinal study on family support and blood glucose management decision-making behavior in patients with gestational diabetes mellitus. Chin J Nurs. (2024) 59:669–76. doi: 10.3761/j.issn.0254-1769.2024.06.003

[9] Zhang X Yin W Liu Q Jin H Zu H . A longitudinal qualitative study on the experience of blood glucose management decision-making in patients with gestational diabetes mellitus. J Nurs. (2023) 30:22–6. doi: 10.16460/j.issn1008-9969.2023.12.022

[10] Yuan L Li Y Jiang X Yang Z . A journey map for improving the cardiac rehabilitation experience and care quality of patients with coronary heart disease: a longitudinal qualitative study. Int J Nurs Stud. (2026) 178:105390. doi: 10.1016/j.ijnurstu.2026.105390 41791180

[11] Jia W Zhang P Zhu D Duolikun N Li H Bao Y . Evaluation of an mHealth-enabled hierarchical diabetes management intervention in primary care in China (ROADMAP): a cluster randomized trial. PloS Med. (2021) 18:e1003754. doi: 10.1371/journal.pmed.1003754 34547030 PMC8454951

[12] Oliveira ESD Presado MH Baixinho CL . The relevance of qualitative research in nursing: exploring experiences and context in care. Rev Bras Enfermagem. (2025) 78Suppl 3:e78supl301. doi: 10.1590/0034-7167.202578supl301 40802378 PMC12334126

[13] Seetharaman S Ekhlaspour L . Revolutionizing OGTT: unlocking the real-time insights and expanded data of continuous glucose monitoring. J Clin Endocrinol Metab. (2025) 110:e3897–8. doi: 10.1210/clinem/dgaf068 39883560 PMC12527421

[14] Merchant T DiTosto JD Gomez-Roas M Williams BR Niznik CM Feinglass J . The role of social support on self-management of gestational diabetes mellitus: a qualitative analysis. J Midwifery Womens Health. (2025) 70:791–9. doi: 10.1111/jmwh.13782 40576167 PMC12354251

[15] Safavi Ardabili N Rafi A Hasannezhad M Aghapour E Neshati A Abdi F . Strategies for the management of gestational diabetes mellitus from the perspective of stakeholders: a systematic review and meta-synthesis. J Diabetes Metab Disord. (2026) 25:34. doi: 10.1007/s40200-025-01848-6 41550249 PMC12804585

[16] P CADA . Classification and diagnosis of diabetes: standards of medical care in diabetes-2022. Diabetes Care. (2022) 45:S17–38. doi: 10.2337/dc22-S002 34964875

[17] Ly S Runacres F Poon P . Journey mapping as a novel approach to healthcare: a qualitative mixed methods study in palliative care. BMC Health Serv Res. (2021) 21:915. doi: 10.1186/s12913-021-06934-y 34479541 PMC8417950

[18] Wang W Hou J Qian P Song T Xie Y . User experience map: a new tool for library user experience librarians. Library Inf Knowledge. (2023) 40:107–17. doi: 10.13366/j.dik.2023.05.107

[19] Lindgren B Lundman B Graneheim UH . Abstraction and interpretation during the qualitative content analysis process. Int J Nurs Stud. (2020) 108:103632. doi: 10.1016/j.ijnurstu.2020.103632 32505813

[20] Korstjens I Moser A . Series: Practical guidance to qualitative research. Part 6: longitudinal qualitative and mixed-methods approaches for longitudinal and complex health themes in primary care research. Eur J Gen Pract. (2022) 28:118–24. doi: 10.1080/13814788.2022.2053106 35593106 PMC9132407

[21] Rogo EJ . Exploring qualitative research. J Dent Hyg. (2024) 98:56–61. 39137996

[22] Carter N Bryant-Lukosius D DiCenso A Blythe J Neville AJ . The use of triangulation in qualitative research. Oncol Nurs Forum. (2014) 41:545–7. doi: 10.1188/14.ONF.545-547 25158659

[23] Olsson S Willman T Hornsten A Otten J Blusi M Lundberg E . Diabetes specialist nurses' experiences of supporting emerging adults living with type 1 diabetes mellitus after the transfer to adult care-a qualitative study. Nurs Res Pract. (2025) 2025:4036033. doi: 10.1155/nrp/4036033 41497566 PMC12767428

[24] Rath K Smitha MV . Gestational diabetes mellitus management among primigravida women: a qualitative study. Cureus J Med Sci. (2025) 17:e88710. doi: 10.7759/cureus.88710 40861653 PMC12375173

[25] Hu G Zhang Z Pu C Shan C Zhu Z Zhou H . A study on the application of peer education based on professional training in patients with gestational diabetes mellitus. Chin J Nurs. (2025) 60:425–32. doi: 10.3761/j.issn.0254-1769.2025.04.006

[26] Shahabi N Kolivand M Salari N Abbasi P . The effect of telenursing training based on family-centered empowerment pattern on compliance with diet regimen in patients with diabetes mellitus type 2: a randomized clinical trial. BMC Endocr Disord. (2022) 22:36. doi: 10.1186/s12902-022-00953-4 35139832 PMC8830007

[27] El Seifi OS Younis FE Ibrahim Y Begum SB Ahmed SF Zayed ES . Telemedicine and gestational diabetes mellitus: systematic review and meta-analysis. Cureus J Med Sci. (2024) 16:e71907. doi: 10.7759/cureus.71907 39564055 PMC11574696

[28] Zhu Y Zhang H Xi Y Zhu H Lu Y Luo X . The implication of diabetes-specialized nurses in aiming for the better treatment and management of patients with diabetes mellitus: a brief narrative review. Diabetes Ther. (2024) 15:917–27. doi: 10.1007/s13300-024-01558-x 38472627 PMC11043239

[29] Ahmadinezhad GS Karimi FZ Abdollahi M NaviPour E . Association between postpartum depression and breastfeeding self-efficacy in mothers: a systematic review and meta-analysis. BMC Pregnancy Childbirth. (2024) 24:273. doi: 10.1186/s12884-024-06465-4 38609849 PMC11015580

[30] Zeinabeh MZ Atefeh A Masumeh GHP Tania D Mojgan S Katayoun A . The effect of mindfulness-based stress reduction counseling on blood glucose and perceived stress in women with gestational diabetes. Rev Bras Ginecologia E Obstetricia. (2023) 45:e517–23. doi: 10.1055/s-0043-1775810 37846184 PMC10579914

[31] American Diabetes Association Professional Practice Committee . Standards of care in diabetes—2025. Diabetes Care. (2025) 48:S1–S270. doi: 10.2337/dc25-S001 39651982

